# Necrotic Liver Metastasis From Anorectal Mucosal Melanoma Mimicking a Pyogenic Liver Abscess: A Case Report

**DOI:** 10.7759/cureus.113077

**Published:** 2026-07-21

**Authors:** Monika Lis, Hubert Piwar, Mateusz Sucharski, Jan Pawlasek, Karolina Hernik

**Affiliations:** 1 Medicine, Masovian Bródnowski Hospital, Warsaw, POL; 2 Medicine, Dr. Anna Gostynska Wolski Hospital, Warsaw, POL; 3 Medicine, Military Institute of Medicine - National Research Institute, Warsaw, POL; 4 Pharmacotherapy and Pharmaceutical Care, Medical University of Warsaw, Warsaw, POL

**Keywords:** anorectal melanoma, hmb-45, liver abscess, liver metastasis, metastatic melanoma, mucosal melanoma, necrotic metastasis, rectal melanoma, sox10

## Abstract

Anorectal mucosal melanoma is a rare and aggressive malignancy that is often diagnosed after regional or distant spread. Liver lesions in patients with melanoma are usually approached with metastatic disease in mind; however, extensive necrosis can make metastases appear clinically and radiologically similar to pyogenic abscesses. We report a 76-year-old woman with a history of anorectal mucosal melanoma treated by abdominoperineal resection with microscopically negative (R0) margins and adjuvant radiotherapy 10 years earlier. She presented with right upper quadrant pain, elevated inflammatory markers, and ultrasound findings interpreted as a hepatic abscess. Empirical broad-spectrum antibiotics and computed tomography (CT)-guided drainage yielded sterile brown hemorrhagic fluid. Persistent symptoms, fever, black-pigmented catheter output, sterile cultures, and magnetic resonance imaging (MRI) showing a centrally necrotic lesion prompted multidisciplinary reassessment. Cytology demonstrated melanin pigment but no viable malignant cells. The patient underwent right hemihepatectomy with cholecystectomy. Histopathology confirmed metastatic melanoma with approximately 95% tumor necrosis and scattered viable cells positive for SRY-box transcription factor 10 (SOX10) and human melanoma black 45 (HMB-45). This case emphasizes that necrotic melanoma metastasis can masquerade as a liver abscess and that sterile cultures, pigmented drainage, prior melanoma history, and discordant imaging should prompt early oncologic reassessment and tissue confirmation.

## Introduction

Anorectal mucosal melanoma is an uncommon melanoma subtype with aggressive biology and poor outcomes. A contemporary systematic review found that anorectal melanoma accounts for less than 1% of all melanomas and approximately 1% of colorectal malignancies; delayed diagnosis is common because bleeding, tenesmus, pruritus, and changes in bowel habits overlap with benign anorectal disease, and 20%-30% of lesions may be amelanotic [1]. In population-based surveillance, epidemiology, and end results (SEER) data, 22% of patients with known stage already had distant metastases at diagnosis, and the estimated five-year survival was 15% [2]. Institutional series and reviews have similarly reported frequent disseminated disease, with the liver, lungs, brain, and nodal basins among recognized metastatic sites [3,4].

Distinguishing a pyogenic liver abscess from necrotic malignancy can be difficult. Hepatic abscesses often present with abdominal pain, fever, and nonspecific inflammatory findings; imaging may show central liquefaction or necrosis, rim enhancement, and diffusion abnormalities, features that can overlap with malignant lesions. This overlap largely reflects shared tissue architecture: both an abscess cavity and a necrotic tumor contain a central zone of liquefied or devitalized tissue surrounded by a vascularized, inflamed rim. On contrast-enhanced computed tomography (CT) and magnetic resonance imaging (MRI), this architecture produces a fluid-like or heterogeneous core with peripheral rim enhancement and may show restricted diffusion on diffusion-weighted MRI [5]. Published reports of melanoma liver metastasis presenting specifically as a liver abscess are scarce. In the closest recent analogue, aspiration of an apparent liver abscess uncovered metastatic rectal mucosal melanoma, with malignant cells partly obscured by necrosis and an inflammation-rich background [6].

We present a patient with a remote history of anorectal mucosal melanoma whose necrotic liver metastasis initially fulfilled clinical and imaging criteria for a pyogenic liver abscess.

## Case presentation

A 76-year-old woman presented to the emergency department with right upper quadrant abdominal pain and loss of appetite. She also reported approximately 6% unintentional loss of body weight over the preceding three months. Her history was notable for histologically confirmed anorectal mucosal melanoma treated 10 years earlier with abdominoperineal resection and permanent colostomy, followed by adjuvant radiotherapy. Operative documentation from the initial melanoma treatment reported microscopically negative (R0) margins. She had also undergone subsequent repair of a parastomal hernia. Her medical history additionally included hypertension, bronchial asthma, recurrent Proteus mirabilis urinary tract infections, and an anxiety disorder. Her regular medications comprised amlodipine, telmisartan, bisoprolol, dexlansoprazole, paroxetine, rupatadine, inhaled fluticasone, fluconazole, and zopiclone. Her family history was negative for malignancy. The case chronology compiled for this report encompasses the original definitive treatment and the present episode; the intervening oncological surveillance period lies outside its scope.

**Figure 1:**
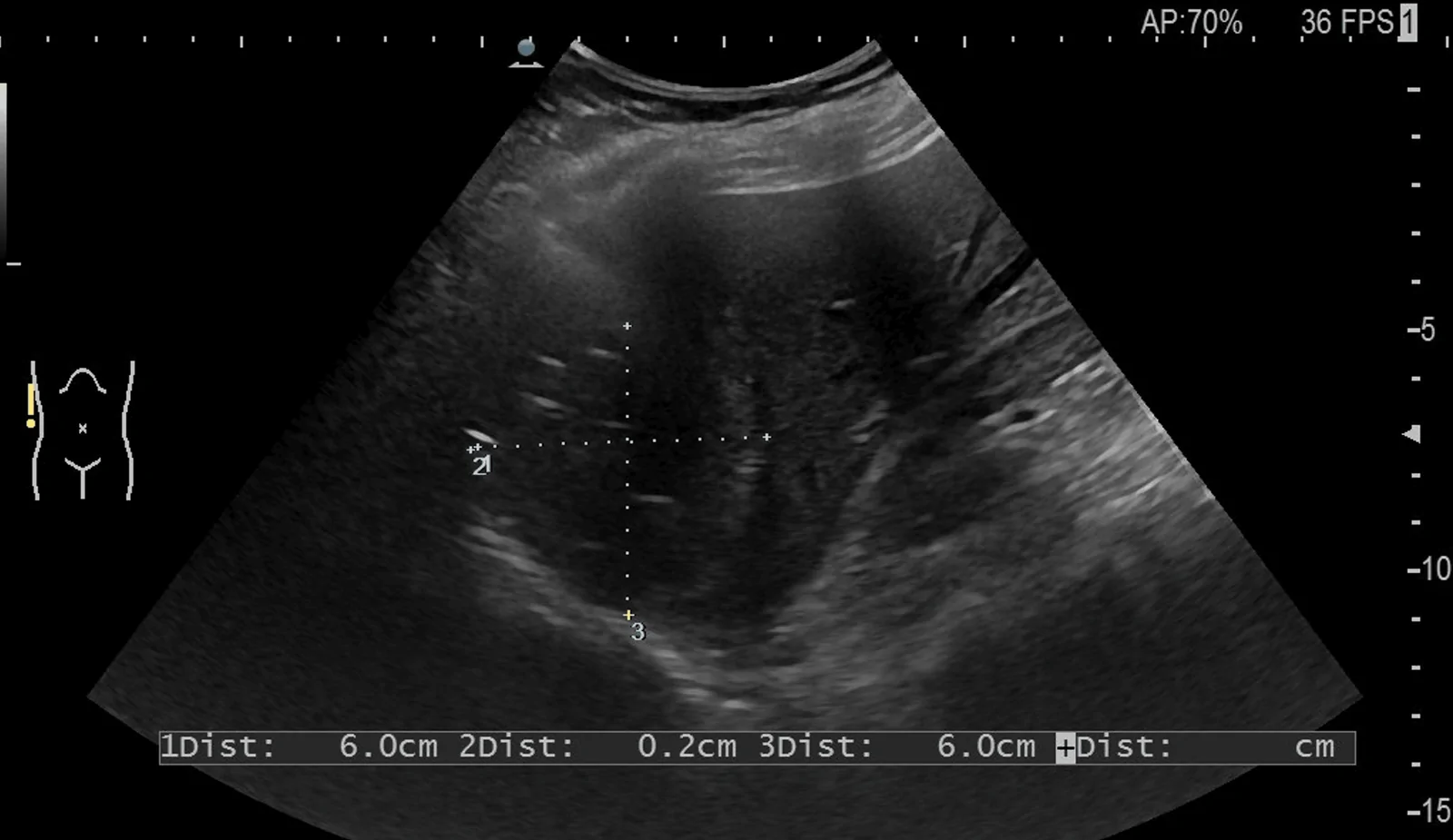
Initial ultrasonography of the right hepatic lobe showing a well-defined, predominantly hypoechoic lesion measuring approximately 60 × 55 × 60 mm, with mixed internal echoes and a peripheral rim The lesion was initially interpreted as a hepatic abscess.

At the referring hospital, abdominal ultrasonography showed a well-defined lesion in the right hepatic lobe measuring approximately 60 × 55 × 60 mm, interpreted as a hepatic abscess (Figure 1). The recorded diagnostic sequence began with ultrasonography, with a pyogenic liver abscess recorded as the imaging-based working diagnosis; the detailed examination on admission for image-guided drainage is reported below. Because transfer for drainage was delayed by local capacity constraints, empirical piperacillin-tazobactam plus levofloxacin was chosen for broad-spectrum coverage of enteric Gram-negative bacilli, anaerobes, and streptococci typically implicated in pyogenic liver abscess, with the fluoroquinolone added for its high biliary and hepatic tissue penetration. The combination was continued for approximately seven days, until the end of the first hospitalization.

On admission for image-guided drainage (case Day 2), the patient was hemodynamically stable: temperature of 36.8°C, blood pressure of 135/73 mmHg, and heart rate of 85 beats/min. Abdominal examination demonstrated right upper quadrant tenderness without peritoneal signs. There was no jaundice or hepatomegaly, and no inflammation was observed around the colostomy.

Laboratory testing showed higher inflammatory markers than in earlier available measurements: the white blood cell count was 12.53 × 10^3^/µL (previously 5.47 × 10^3^/µL), the neutrophil count was 11.09 × 10^3^/µL (previously 3.36 × 10^3^/µL), and C-reactive protein was 115 mg/L (previously 57.2 mg/L). There was mild normocytic anemia (hemoglobin, 9.6 g/dL; previous value, 9.9 g/dL) and thrombocytosis (platelet count, 606 × 10^3^/µL). Alkaline phosphatase was 183 U/L and gamma-glutamyl transferase was 219 U/L, whereas alanine aminotransferase, aspartate aminotransferase, albumin, bilirubin, and creatinine were within their respective reference ranges. The available laboratory values and reference ranges are summarized in Table 1.

**Table 1:** Laboratory values across the clinical course, with corresponding reference ranges A dash (—) indicates that no result was reported at that time point. Abbreviations: CKD-EPI, Chronic Kidney Disease Epidemiology Collaboration; eGFR, estimated glomerular filtration rate.

Parameter	Reference range	Earlier available value	Drainage admission (Day 2)	First discharge (Day 9)	Readmission (Day 14)
White blood cell count, ×10³/µL	4-10	5.47	12.53	5.91	7.59
Neutrophils, ×10³/µL	1.5-7.5	3.36	11.09	3.29	5.19
C-reactive protein, mg/L	<10	57.2	115	61.3	125.8
Hemoglobin, g/dL	12-16	9.9	9.6	11.1	11.2
Mean corpuscular volume, fL	80-97	—	88.8	88.2	87.7
Hematocrit, %	37-47	—	30	33	34
Platelets, ×10³/µL	150-400	—	606	—	443
Alkaline phosphatase, U/L	30-120	—	183	—	341
Gamma-glutamyl transferase, U/L	7-50	—	219	—	166
Total bilirubin, mg/dL	0.2-1.2	—	0.28	—	0.59
Alanine aminotransferase, U/L	7-30	—	25	—	15
Aspartate aminotransferase, U/L	5-40	—	20	—	18
Albumin, g/dL	3.5-5.3	—	4.3	—	4.1
Amylase, U/L	28-100	—	19	—	22
Urea, mg/dL	15-48	—	18	14	15
Creatinine, mg/dL	0.5-1.0	—	0.66	—	0.57
eGFR (CKD-EPI), mL/min/1.73 m²	>90	—	61	—	90

Subsequently, CT-guided drainage yielded approximately 45 mL of brown fluid resembling hemolyzed blood. Because the lesion was predominantly liquefied, an attempt to obtain sufficient solid material for core biopsy was unsuccessful; only aspirated fluid was therefore submitted for microbiological and cytological analysis. Gram and fungal stains were negative. Aerobic cultures were reviewed at 48 hours, anaerobic and slow-growing cultures were incubated for at least four days, and the final report at five days showed no growth. Cytology revealed abundant melanin pigment and a few cells positive for cluster of differentiation 68 (CD68); SRY-box transcription factor 10 (SOX10) and pancytokeratin (AE1/AE3) staining were negative. No definitive malignant cells were identified. The patient was discharged in good condition on case Day 9 with an indwelling pigtail drain, which produced 50-100 mL daily.

**Figure 2:**
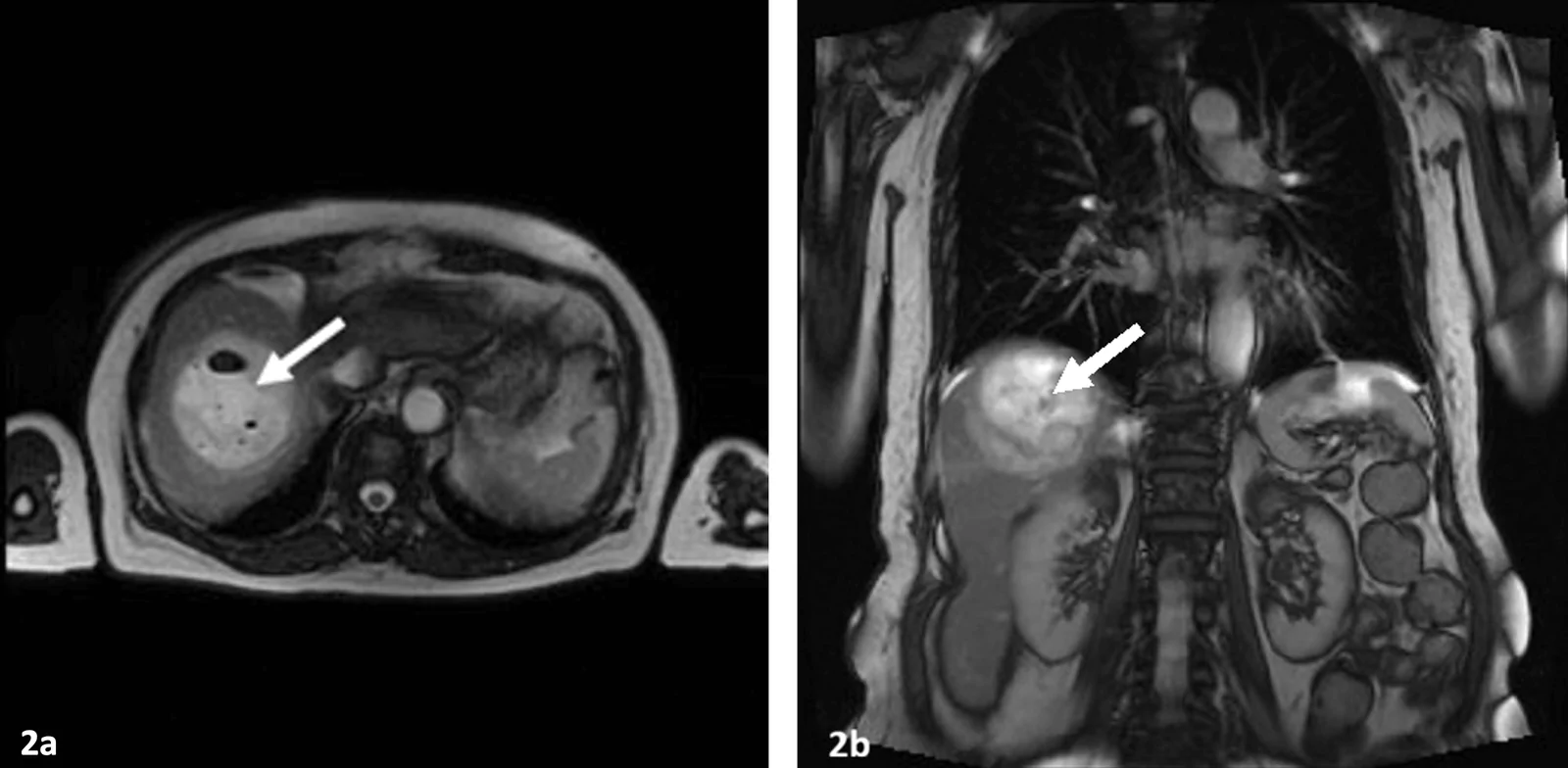
T2-weighted magnetic resonance images of the right hepatic lobe lesion The arrows indicate a heterogeneous lesion with central hyperintensity corresponding to extensive central necrosis in the clinical context of metastatic melanoma.

On case Day 14, five days after discharge, she was readmitted because of worsening abdominal pain, now localized to the left lower quadrant at the site of her permanent colostomy and previous parastomal hernia repair, fever to 38.7°C, chills, and increased drain output to approximately 150 mL/day with black discoloration of the fluid. No specific cause was established for the left-sided pain. Repeat ultrasonography showed enlargement of the collection. Subsequent magnetic resonance imaging (MRI) demonstrated a 64 × 72 × 66 mm centrally necrotic lesion in the right hepatic lobe with features suspicious for melanoma metastasis, as well as a suspicious lesion at the first thoracic vertebra (Figure 2). Fluorodeoxyglucose positron emission tomography/CT (FDG-PET/CT) was not performed because local access was very limited and waiting times were long. The T1 vertebral lesion remained indeterminate during the documented diagnostic pathway. Repeat laboratory testing showed persistent inflammation with worsening cholestasis (C-reactive protein, 125.8 mg/L; alkaline phosphatase, 341 U/L) after a transient improvement during the first admission (Table 1). Repeat cytology again showed melanin pigment in the drained material, but no viable malignant cells. Given the culture-negative aspirate, black-pigmented drainage, prior melanoma history, and MRI findings, the multidisciplinary oncology team recommended operative management.

On case Day 36, 35 days after the initial presentation, the patient underwent a classical open right hemihepatectomy involving hepatic segments V, VI, VII, and VIII, with cholecystectomy as part of the formal procedure. The operation lasted approximately 3 hours and 54 minutes, with an estimated blood loss of 650 mL.

Intraoperatively, after standard preparation and draping, the parietal peritoneum of the right upper quadrant, the right diaphragmatic dome, and the greater omentum were found to be stained with black pigment, and a partial omentectomy was performed; there were no other visible signs of peritoneal dissemination. A soft tumor of approximately 10 cm, entirely covered by liver parenchyma, was palpable in segments VI/VII, and the previously placed pigtail drain was removed. The right hemiliver was mobilized, and its ligamentous attachments were divided; the inferior vena cava was dissected from the posterior surface of the liver, and the hepatocaval ligament was divided. Cholecystectomy was performed, and the right hepatic vein was encircled. An anatomical right hemihepatectomy was completed with a margin of approximately 1-2 cm of uninvolved parenchyma; the vascular and biliary pedicles were secured on the transection plane with clips and ligatures, and the right hepatic vein was clamped and oversewn with a continuous suture. The cut liver surface was treated with argon-beam coagulation, tissue sealant, a fibrin sealant patch, and oxidized cellulose. After confirmation of hemostasis and copious saline lavage, two drains were placed beneath the right diaphragmatic dome through a separate incision, correct instrument and swab counts were confirmed, and the abdominal wall was closed in layers.

Macroscopic examination of the resected right hemiliver (20.5 × 10 × 10 cm) revealed a well-circumscribed, soft, brown tumor measuring 9 × 7.5 × 5.5 cm with extensive central necrosis. Histopathologic examination showed extensive necrosis involving approximately 95% of the lesion, with numerous cells containing melanin pigment. The gallbladder (8.5 cm long, 3 cm in diameter) was thin-walled and unremarkable, and a separately submitted segment of greater omentum (28 × 8 × 1.3 cm) showed multiple petechiae without focal lesions. Immunohistochemistry was positive for SOX10 and human melanoma black 45 (HMB-45) in scattered viable tumor cells. Metastatic infiltration was present in adjacent adipose tissue. The resection margin was less than 3 cm but microscopically negative (R0).

The early postoperative course was notable for a fall in hemoglobin to 7.6 g/dL on postoperative Day 2 (from 10 g/dL on the day of surgery). Two units of packed red blood cells were transfused without transfusion-related complications, and a post-transfusion complete blood count showed an increase in hemoglobin to 9.6 g/dL. The two drains yielded a combined 150 mL of serosanguineous fluid on postoperative Day 1 and were removed on postoperative Day 6. A postoperative urinary tract infection was managed conservatively during the surgical admission. Wound healing was satisfactory. The postoperative follow-up reported here extends through postoperative Day 13 (case Day 49), when the patient was referred to oncology for further care and molecular/genetic evaluation. Later oncological care was beyond the follow-up period of this report. The major clinical events are summarized in Table 2.

**Table 2:** Chronology of major clinical events Case days were counted from the initial presentation (Day 1), and postoperative days were counted from the day of surgery. Events are presented in chronological order.

Clinical event	Timing
Initial presentation and admission; empirical antibiotics started	Day 1
Admission for image-guided drainage	Day 2
CT-guided percutaneous drainage; pigtail catheter placed	After admission for drainage
Discharge with pigtail catheter	Day 9
Readmission with worsening pain, fever, and black drain output	Day 14
Repeat ultrasonography and MRI	After readmission
Multidisciplinary decision for surgery	After repeat imaging
Right hemihepatectomy with cholecystectomy	Day 36
End of surgical-ward follow-up; oncology referral	Day 49 (postoperative Day 13)

## Discussion

This case illustrates a diagnostic trap: a necrotic melanoma metastasis may satisfy the initial clinical and radiological expectations for a pyogenic liver abscess. At the first presentation, the patient had abdominal pain, elevated inflammatory markers, and a cystic-appearing hepatic lesion. However, several findings were atypical for an uncomplicated bacterial abscess: a culture-negative aspirate, brown-to-black drainage, melanin pigment in cytology, and a history of mucosal melanoma. MRI reviews emphasize that abscesses and malignancies can share central necrosis, rim enhancement, and diffusion abnormalities; therefore, imaging should be interpreted together with the clinical course, microbiology, and tissue findings [5].

The differential diagnosis of a cystic or necrotic hepatic lesion is broad and includes pyogenic and amoebic abscess, a simple or complicated hemorrhagic cyst, hydatid disease, primary hepatic malignancy, and necrotic metastases from gastrointestinal, neuroendocrine, or melanocytic primaries [5]. Conversely, a pyogenic liver abscess as the sentinel presentation of an occult malignancy has been reported, further supporting histological confirmation when the course is atypical [7]. In a patient with a prior malignancy, a metastasis with cystic or necrotic degeneration must remain in the differential even when inflammatory markers and imaging initially favor infection, as this case illustrates.

The case reported by Ng et al. is particularly relevant because metastatic rectal mucosal melanoma presented as a liver abscess aspirate; atypical malignant cells were difficult to recognize against an inflammation-rich and necrotic background, and melanocytic immunostaining was required for confirmation [6]. Our case goes one step further diagnostically: two drainage cytology samples showed melanin pigment, but no viable malignant cells, and definitive diagnosis was possible only after resection of a largely necrotic mass. This explains why pigmented, culture-negative drainage should not be dismissed as a contaminated or hemorrhagic abscess when the overall clinical picture remains discordant.

Anorectal melanoma is also prone to diagnostic delay at the primary site because it can resemble benign anorectal conditions. Felz et al. described four cases initially treated as hemorrhoids, each ultimately fatal with widely metastatic disease; they emphasized that apparent hemorrhoids that enlarge, are pigmented, ulcerated, indurated, or remain refractory to conservative therapy should be evaluated for melanoma [8]. Akar similarly reported anal melanoma with multiple liver metastases after a previous diagnosis of thrombosed external hemorrhoids [9]. Although the primary tumor in our patient had already been treated with R0 resection, this history became the key clue when the presumed abscess behaved atypically.

Surgical management of primary anorectal melanoma remains debated. Retrospective studies and reviews have not demonstrated a consistent overall survival advantage for abdominoperineal resection over wide local excision, although more radical surgery may improve local control in selected patients [4,10-13]. Because radical surgery has not translated into a survival benefit, sphincter-preserving wide local excision with clear margins is generally favored when feasible, with radiotherapy used for local control in selected cases [10,11,13]. The present patient posed a different question: how to manage a symptomatic, enlarging, culture-negative, centrally necrotic liver lesion with repeated nondiagnostic cytology and a plausible melanoma history. In this setting, multidisciplinary reassessment was critical. Hepatectomy provided both definitive diagnosis and complete macroscopic treatment of the suspicious lesion, but this case should not be read as evidence that hepatic resection is standard for all melanoma liver metastases. Rather, it supports individualized decision-making when presumed abscess management fails and malignancy remains likely. A systematic review of historical, highly selected surgical series, largely comprising ocular and cutaneous melanoma, reported a pooled median overall survival of approximately 24 months and a five-year survival of approximately 24% after hepatic resection [14]. In the present case, however, the suspicious T1 vertebral lesion remained indeterminate; consequently, liver-confined disease could not be confirmed. Additional case reports of rectal or anorectal melanoma with hepatic and multiorgan metastases, although not abscess-mimicking presentations, further support keeping metastatic melanoma in the differential diagnosis when a patient with melanoma history develops suspicious hepatic lesions [15,16].

The biology of anorectal mucosal melanoma may influence systemic treatment selection once metastatic disease is confirmed. In the systematic review by Paolino et al., molecular results were available only for small subsets: B-Raf proto-oncogene, serine/threonine kinase (BRAF) mutations in one of 19 tested tumors, KIT alterations in 11 of 23, and a neuroblastoma RAS viral oncogene homolog (NRAS) mutation in one of six [1]. These small denominators preclude precise prevalence estimates. In a pooled analysis, the programmed cell death protein 1 (PD-1) inhibitor nivolumab, alone or combined with the cytotoxic T-lymphocyte-associated protein 4 (CTLA-4) inhibitor ipilimumab, showed activity in advanced mucosal melanoma, although objective response rates and median progression-free survival were numerically lower than in cutaneous melanoma [17]. Imatinib has shown activity in a subset of melanomas harboring functionally relevant KIT alterations [18]. Patient-specific molecular profiling and serum lactate dehydrogenase assessment were not part of the perioperative episode. Referral for molecular/genetic evaluation and oncological follow-up was arranged after R0 resection; subsequent systemic-treatment planning belonged to that next phase of care.

The practical lesson is to avoid diagnostic anchoring. In a patient with a prior melanoma, especially mucosal melanoma, a culture-negative hepatic collection with pigmented or hemorrhagic drainage should trigger early reassessment, repeat tissue sampling or core biopsy when feasible, and close collaboration among surgery, radiology, pathology, infectious diseases, and oncology.

## Conclusions

Necrotic liver metastasis from anorectal mucosal melanoma can closely mimic a pyogenic liver abscess. A culture-negative aspirate, pigmented drainage, melanin pigment on cytology, atypical MRI features, and a history of melanoma are important warning signs.

Non-diagnostic aspirate cytology does not exclude melanoma when necrosis is extensive. Early multidisciplinary reassessment and definitive tissue diagnosis are essential to prevent prolonged treatment as infection alone and to guide timely oncological management and molecular characterization.
